# Long-Term Inhalation of Ultrafine Zinc Particles Deteriorated Cardiac and Cardiovascular Functions in Rats of Myocardial Infarction

**DOI:** 10.3389/fphys.2022.921764

**Published:** 2022-07-13

**Authors:** Yunlong Huo, Li Li

**Affiliations:** Institute of Mechanobiology & Medical Engineering, School of Life Sciences and Biotechnology, Shanghai Jiao Tong University, Shanghai, China; PKU-HKUST Shenzhen-Hong Kong Institution, Shenzhen, China; Department of Mechanics and Engineering Science, College of Engineering, Peking University, Beijing, China

**Keywords:** myocardium infraction, speckle-tracing echocardiography, strain analysis, ultrafine zinc particle, Womersley analysis

## Abstract

Substantial ultrafine zinc particles exist in air pollutions. The level of Zn concentrations in serum and tissue could affect patients with myocardial infarction (MI). The aim of the study is to investigate the change of cardiac functions and peripheral hemodynamics in MI rats after long-term inhalation of ultrafine Zn particles. Coronary artery ligation surgery was performed to induce MI in Wistar rats. The inhalation of ultrafine Zn particles was carried out for 6 weeks after the operation. Physiological and hemodynamic measurements and computational biomechanics analysis were demonstrated in eight groups of rats at postoperative 4 and 6 weeks. There was no statistical significance between shams and shams with inhalation of ultrafine Zn particles. There were significant impairments of cardiac and hemodynamic functions in MI rats. In comparison with MI rats, the inhalation of ultrafine Zn particles for 4 weeks slowed down the progression from MI to heart failure, but the inhalation for 6 weeks accelerated the process. The long-term inhalation of ultrafine zinc particles induced excessive accumulation of zinc in serum and tissue, which deteriorated cardiac and hemodynamic dysfunctions in MI rats. The findings suggested the importance for regulating Zn intake of MI patients as well as looking at ways to lower zinc concentrations in air pollutions.

## Introduction

The American Heart Association reports long-term exposure to particle matters (PMs) in air pollution resulting to cardiovascular morbidity and mortality (Robert, 2004). In comparison with fine particle pollution (PM2.5), ultrafine particles (UFPs) (PM0.1) produced stronger chemical reaction given its small volume and large surface area (Andre 2006; Franck et al., 2011; Karottkiet al., 2014), which could increase vascular tension of systemic circulation resulting in high blood pressure (Mills et al., 2005; Törnqvist et al., 2007) as well as impair regulation of endogenous fibrinolysis accelerating the process of myocardial remodeling and fibrosis (Wold et al., 2012). Hence, UFPs in air pollution should be more detrimental to patients with myocardial infarction (MI) (Andre, 2006; Franck, et al., 2011; Karottki et al., 2014).

UFPs contained substantial airborne trace metals (Wolfram 2006). The concentrations of trace metals were in the order of Zn > Pb > Cu > Cr > V > Ni in four cities of the Yangtze River Delta (YRD) Metropolitan Area, China, i.e., Shanghai, Nanjing, Hangzhou, Ningbo (Ming et al., 2017). Because of higher concentration of Zn in air pollution, we have investigated the effects of short-term (4 weeks) inhalation of ultrafine Zn particles (wrapped by a layer of ZnO) on MI rats, which was found to alleviate cardiac and hemodynamic dysfunctions in disagreement with the assumption of toxicity hazards (Li et al., 2021). Hence, it is required to further investigate the effects of long-term inhalation of ultrafine zinc particles (wrapped by a layer of ZnO) on MI rats. The words “wrapped by a layer of ZnO” are neglected in the following text unless specifically noted.

The objective of the study is to investigate the change of cardiac functions and peripheral hemodynamics in MI rats after long-term inhalation of ultrafine Zn particles. Here, we hypothesized that the toxic effect of ultrafine Zn particles on the progression from MI to heart failure (HF) is associated with time interval of inhaling the particles, i.e., short-term inhalation of ultrafine Zn particles inhibits the progression, but long-term inhalation deteriorates it. To test the hypothesis, Wistar rats were used for coronary artery ligation surgery to induce MI. Partial shams and MI rats underwent inhalation control of ultrafine Zn particles for 6 weeks. Physiological and hemodynamic measurements were demonstrated in the LV and carotid artery for 4 and 6 weeks after the ligation surgery. The speckle tracking echocardiography (STE) was used to analyze LV functions. The Windkessel model was performed for the hemodynamic analysis in the carotid artery.

## Methods

### Experimental Measurements

Wistar male rats (Beijing Vital River Laboratory Animal Technology) were used in the study. All animals (6 weeks) were housed at standard SPF laboratory and free access to standard rodent chow and water. Myocardial infarction was induced by the left anterior descending (LAD) artery ligation surgery, where a 7-0 suture was ligated at ∼1 mm position distal to the LAD artery under the tip of the left auricle (Brenner et al., 2004; Gao et al., 2010), which was considered successful when the LV anterior wall became pale. Alternatively, the ligation suture was placed in the LAD artery, but removed in sham-operated animals. There were four groups: sham group (Sham), sham with inhalation of ultrafine Zn particles (ShamZn), myocardial infarction group (MI), and MI with inhalation of ultrafine Zn particles (MIZn). Three days after the surgery, ShamZn and MIZn groups were exposed in the environment filled with ultrafine zinc particle (diameter of 50 nm and density of 500 μg/m^3^, Beijing Deke Daojin Science and Technology Co., Ltd.) (Bing et al., 2020). MIZn and ShamZn groups inhaled ultrafine Zn particles for 4 h per day and 4 days per week for 4 and 6 weeks postoperatively (Li et al., 2021). Sixty rats underwent the LAD ligation surgery and eight rats were dead immediately after the surgery, the rest of which were divided into MI and MIZn groups of 26 each. There were four and three dead animals in MI and MIZn groups at postoperative 4 weeks (4W) and subsequently four dead animals in each group at postoperative 6 weeks (6W). There were no dead animals in Sham and ShamZn groups. The four groups were further divided into two subgroups, i.e., postoperative 4 weeks (4W) and 6 weeks (6W): Sham4 (*n* = 8), ShamZn4 (*n* = 8), MI4 (*n* = 10), MIZn4 (*n* = 11), Sham6 (*n* = 8), ShamZn6 (*n* = 8), MI6 (*n* = 8), and MIZn6 (*n* = 8). Echocardiographic measurements of animal hearts (all animals) were carried out under anesthesia for 4 and 6 weeks postoperatively, based on which myocardial deformation measurements were demonstrated with advanced STE (Niu et al., 2020). Hemodynamic measurements (all animals) were consistent with those in a previous study (Bing et al., 2020). Histological evaluation and Zn detection (*n* = 6 in each group) were described in the Appendix. All experiments were performed in accordance with Chinese National and Peking University ethical guidelines regarding the use of animals in research, consistent with the NIH guidelines (Guide for the care and use of laboratory animals) on the protection of animals used for scientific purposes. The experimental protocol was approved by the Animal Care and Use Committee of Peking University, China.

### Mathematic Method

Based on pressure and flow waves of carotid artery, the time-averaged pressure and flow over a cardiac cycle (P_mean_ and Q_mean_) are computed consistent with previous studies (Bing et al., 2020). The cardiac output (CO), equal to Q_mean_ × 60 s. The arterial tree was modeled as an elastic chamber (Windkessel) with total compliance, C, and peripheral resistance, R ( $\approx P_{ao,mean}/Q_{mean}$ , where $P_{ao,mean}$ is the mean aortic pressure). In the diastolic period, the blood pressure decays with a power form:

$$p\left(t\right)=p_{1}\times e^{-\frac{t}{R\times C}}$$ (1)

where $p_{1}$ is the peak blood pressure at the time $t_{1}$ . Taking the natural log function, Eq. 1 can be written as:

$$\ln p\left(t\right)=-\frac{t}{R\times C}+\ln p_{1}$$ (2)

Provided the slope of k between ln⁡p(t) and *t*, total compliance, C, is obtained:

$$\text{C}=-\frac{1}{R\times k}$$ (3)

These equations are used to solve the total compliance and peripheral resistance.

### Statistical Analysis

Experimental measurements were repeated 3 times and averaged per animal. All parameters were presented as mean ± SEM by averaging over all animals in each group. The two-way ANOVA (SigmaStat3.5) was used to detect the statistical difference of morphometric and hemodynamic parameters between sham and MI groups and between inhalation of zinc particle and no inhalation groups, where *p* < 0.05 was indicative of a significant difference between the two populations.

## Results

**FIGURE 1:**
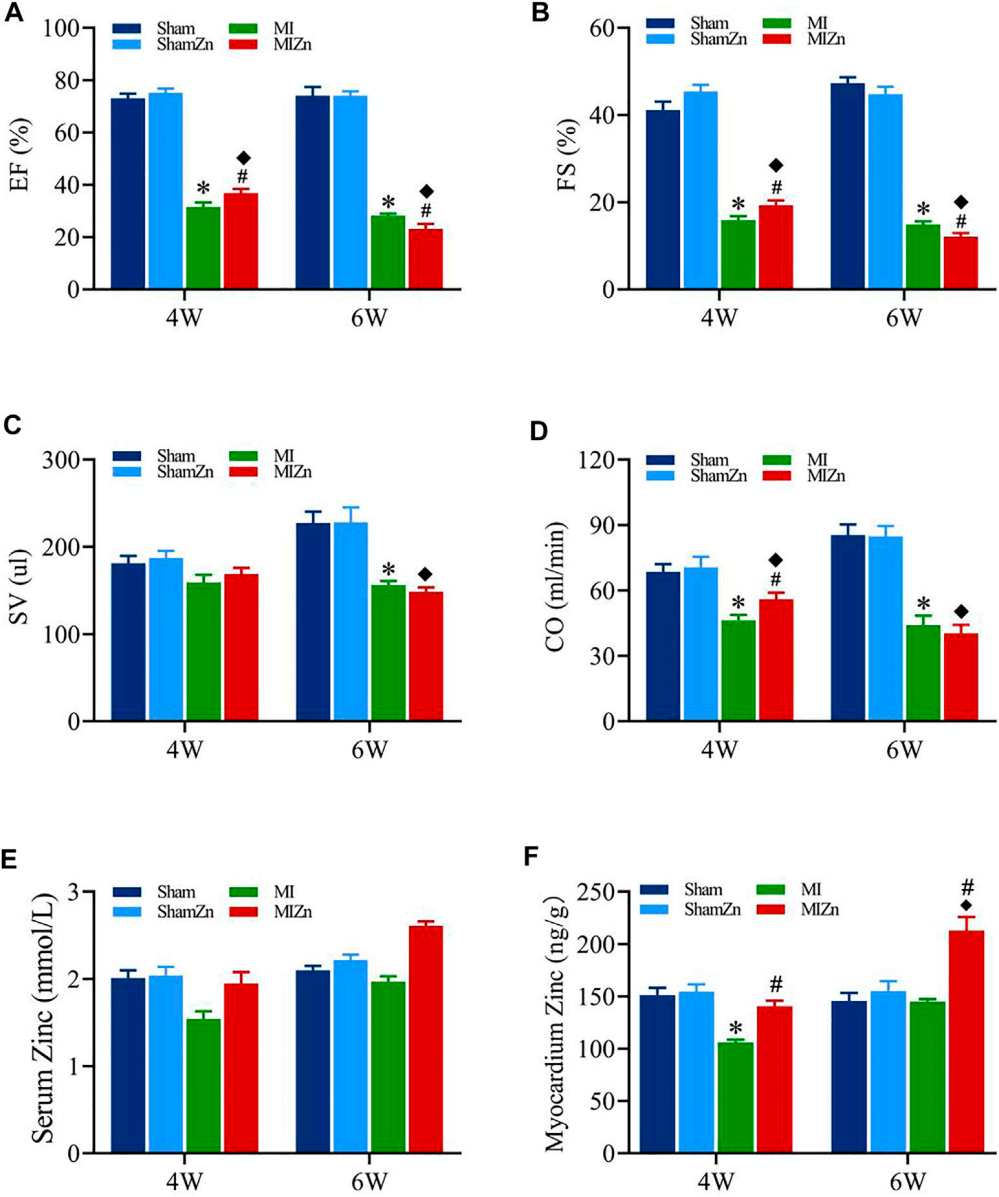
Cardiac functions and zinc levels in eight groups. Panels **(A–D)** show the change of EF, FS, SV, CO, respectively. Panels **(E,F)** show the change of serum and myocardium zinc concentrations, respectively. All of the data were shown as Mean ± SEM. ^*^ *p* < 0.05, MI vs. Sham; ^◆^ *p* < 0.05, ShamZn vs. MIZn; ^#^ *p* < 0.05, MIZn vs. MI.

Figure 1 shows cardiac functions and zinc levels in eight groups, where EF, FS, SV and CO decrease significantly in MI groups despite of no statistical difference in sham groups. The EF and FS values in the MI4 group are lower than the MIZn4 group (EF/FS: 31.48%/15.87 % vs. 36.76%/19.28%) statistically, but those in the MI6 group are higher than the MIZn6 group (EF/FS: 28.12%/14.85% vs. 23.1%/12.07%) (Figures 1A,B). The SV and CO show similar changes (Figures 1C,D). The zinc levels are reduced (25%–35% in serum and myocardium) by the MI at post-operative 4 weeks and recover to normal level at post-operative 6 weeks. The inhalation of ultrafine zinc particles retains normal zinc levels in the MIZn4 group and increases the zinc level significantly (25%–35% in serum and myocardium) in the MIZn6 group. The inhalation of ultrafine zinc particles has no effects on cardiac functions and zinc levels in healthy rats, as shown in Figures 1E,F. Table 1 lists morphometric parameters in the LV of eight groups at systole and diastole. The anterior wall of the LV is significantly reduced in MI rats because of myocardial necrosis while the LV volume is increased. The LVAW in the MIZn4 group is higher than the MI4 group, but the LVAW in the MIZn6 group is significantly lower than the MI6 group. In contrast, ESV and EDV in the MIZn4 group are lower than the MI4 group, but the values in the MIZn6 group are significantly higher than the MI6 group.

**TABLE 1:** Morphometric parameters in the LV of eight groups at systole and diastole.

Groups	LVAW;s (mm)	LVAW;d (mm)	LVPW;s (mm)	LVPW;d (mm)	LVID;s (mm)	LVID;d (mm)	ESV (µL)	EDV (µL)
Sham4	2.69 ± 0.07	1.67 ± 0.04	2.62 ± 0.04	1.71 ± 0.04	3.87 ± 0.11	6.40 ± 0.08	67.00 ± 3.84	224.3 ± 13.7
ShamZn4	2.74 ± 0.06	1.89 ± 0.02	2.90 ± 0.09	1.90 ± 0.05	3.77 ± 0.13	6.90 ± 0.16	68.62 ± 3.51	238.4 ± 12.76
MI4	1.07 ± 0.07^*^^*^ *p < 0.0001*	1.05 ± 0.07^*^^*^ *p < 0.0001*	2.61 ± 0.16 ^*^ *p > 0.9999*	1.91 ± 0.06 ^*^ *p = 0.2396*	8.02 ± 0.27^*^^*^ *p < 0.0001*	10.06 ± 0.26^*^^*^ *p < 0.0001*	383 ± 10.71^*^^*^ *p < 0.0001*	565 ± 22.77^*^^*^ *p < 0.0001*
MIZn4	1.16 ± 0.04^◆^^◆^ *p < 0.0001* ^#^ *p = 0.9918*	1.20 ± 0.10^◆^^◆^ *p < 0.0001* ^#^ *p = 0.8096*	2.62 ± 0.11 ^◆^ *p = 0.6435* ^#^ *p > 0.9999*	1.93 ± 0.08 ^◆^ *p > 0.9999* ^#^ *p > 0.9999*	7.26 ± 0.23^◆#^^◆^ *p < 0.0001* ^#^ *p = 0.0467*	9.48 ± 0.32^◆^^◆^ *< 0.0001* ^#^ *p = 0.5974*	352 ± 6.81^◆#^^◆^ *p < 0.0001* ^#^ *p = 0.0209*	493 ± 22.83^◆#^^◆^ *p < 0.0001* ^#^ *p = 0.0318*
Sham6	2.96 ± 0.18	2.01 ± 0.08	3.29 ± 0.13	2.13 ± 0.08	4.17 ± 0.14	7.23 ± 0.21	75.85 ± 5.76	288.00 ± 13.96
ShamZn6	3.06 ± 0.04	1.95 ± 0.03	3.34 ± 0.13	2.12 ± 0.07	4.45 ± 0.15	7.59 ± 0.28	75.92 ± 5.29	309.70 ± 24.84
MI6	0.93 ± 0.07^*^^*^ *p < 0.0001*	1.05 ± 0.11^*^^*^ *p < 0.0001*	2.84 ± 0.11 ^*^ *p = 0.1801*	2.21 ± 0.07 ^*^ *p = 0.9887*	8.26 ± 0.23^*^^*^ *p < 0.0001*	10.28 ± 0.38^*^^*^ *p < 0.0001*	402 ± 7.57^*^^*^ *p < 0.0001*	609 ± 12.39^*^^*^ *p < 0.0001*
MIZn6	0.71 ± 0.07^◆#^^◆^ *p < 0.0001* ^#^ *p = 0.0485*	0.69 ± 0.08^◆#^^◆^ *p < 0.0001* ^#^ *p = 0.0323*	2.71 ± 0.15^◆^^◆^ *p = 0.0185* ^#^ *p = 0.9908*	2.05 ± 0.06 ^◆^ *p = 0.9991* ^#^ *p = 0.7576*	9.05 ± 0.18^◆#^^◆^ *p < 0.0001* ^#^ *p = 0.0189*	10.89 ± 0.24^◆^^◆^ *p < 0.0001* ^#^ *p = 0.7027*	438 ± 14.02^◆#^^◆^ *p < 0.0001* ^#^ *p = 0.0494*	647 ± 10.39^◆#^^◆^ *p < 0.0001* ^#^ *p = 0.0319*

All of the data were shown as Mean ± SEM. ^*^ *p* < 0.05, MI vs. Sham; ^◆^ *p* < 0.05, ShamZn vs. MIZn; ^#^ *p* < 0.05, MIZn vs. MI.

**FIGURE 2:**
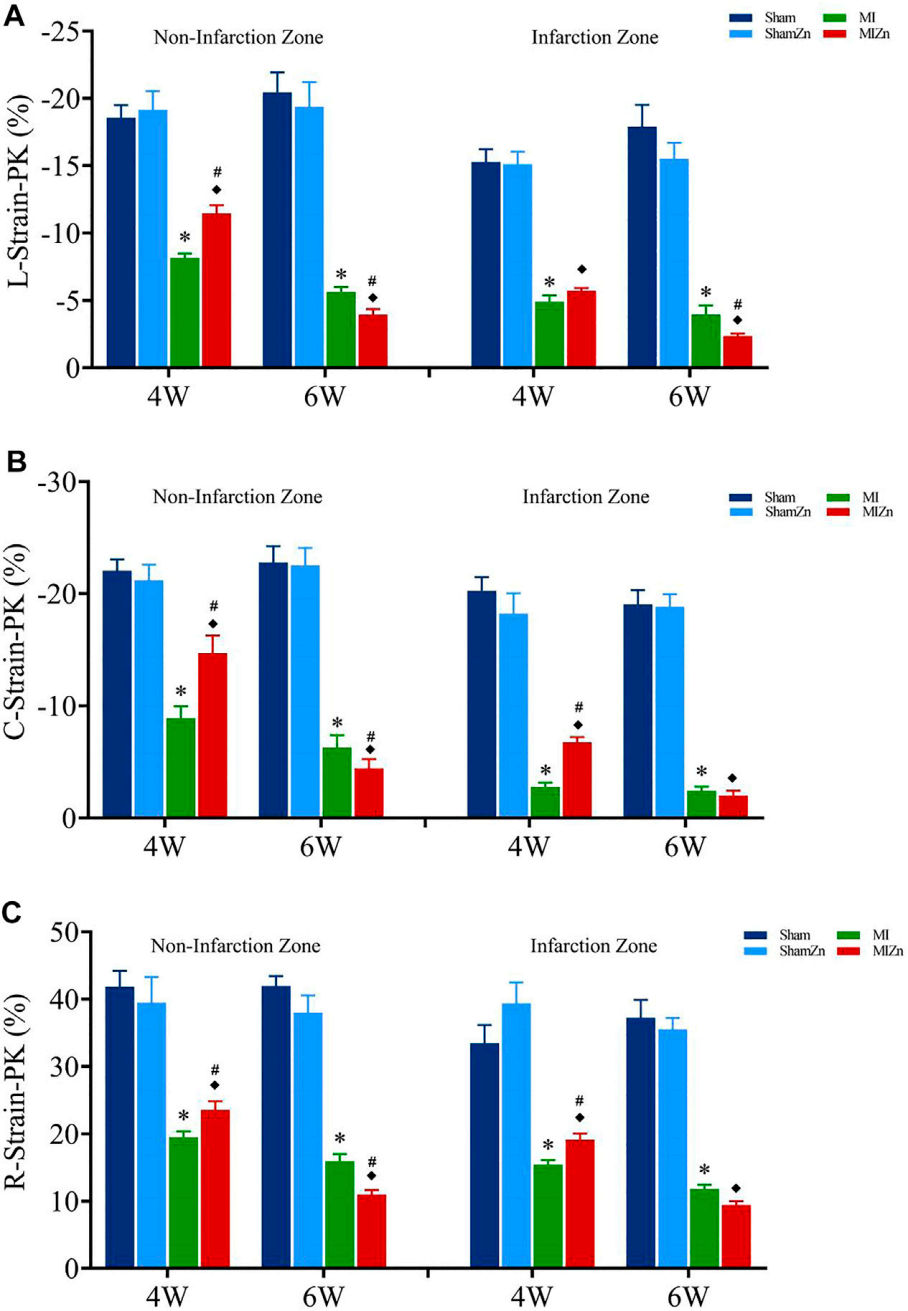
Strain peak values in the longitudinal **(A)**, circumferential **(B)** and radial **(C)** directions of myocardial infarction zone and non-infarction zone in eight groups, L: Longitudinal, C: Circumferential, R: Radial. All of the data were shown as Mean ± SEM. ^*^ *p* < 0.05, MI vs. Sham; ^◆^ *p* < 0.05, ShamZn vs. MIZn; ^#^ *p* < 0.05, MIZn vs. MI.

**FIGURE 3:**
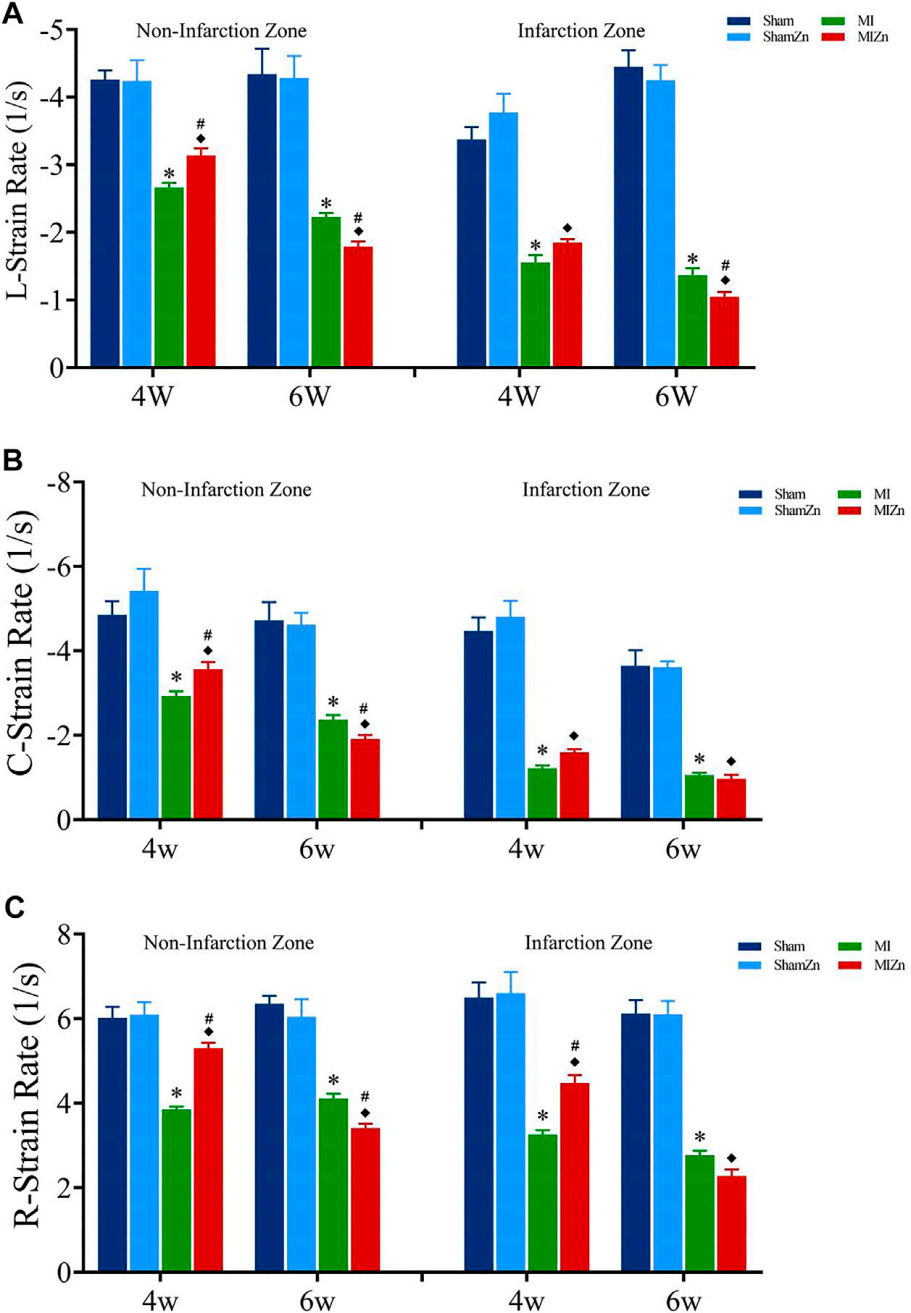
Strain rate peak values in the longitudinal **(A)**, circumferential **(B)** and radial **(C)** directions of myocardial infarction zone and non-infarction zone in eight groups, L: Longitudinal, C: Circumferential, R: Radial. All of the data were shown as Mean ± SEM. ^*^ *p* < 0.05, MI vs. Sham; ^◆^ *p* < 0.05, ShamZn vs. MIZn; ^#^ *p* < 0.05, MIZn vs. MI.

Figures 2, 3 show peak values of strains and strain rates, respectively, in the longitudinal, circumferential and radial directions of myocardial infarction and non-infarction zones in eight groups. There is no statistical difference between Sham and ShamZn groups at postoperative 4 and 6 weeks. In comparison with Sham and ShamZn groups, peak values of strains and strain rates in both infarction and normal regions are significantly reduced in MI and MIZn groups. Peak values in the three directions in the MIZn4 group are higher than those in the MI4 group. In contrast, peak values of strains and strain rates in the MIZn6 group are lower than those in the MI6 group.

Morphological and hemodynamic parameters of the right carotid artery are listed in Table 2. There is no statistical difference between Sham and ShamZn groups at postoperative 4 and 6 weeks. Myocardial infarction deteriorates hemodynamic environment in peripheral arteries significantly. The short-term (4 weeks) inhalation of ultrafine zinc particles inhibits the impairments caused by the MI (Resistances in MI4 vs. MIZn4: 280 vs. 196, *p* < 0.005). On the other hand, the long-term (6 weeks) inhalation of ultrafine zinc particles accelerates the impairments (Resistances in MI6 vs. MIZn6: 339 vs. 409, *p* < 0.005).

**TABLE 2:** Morphological and hemodynamic parameters in the right carotid artery of eight groups.

Groups	Diameter (mm)	CAS (mm^2^)	Flow velocity (m/s)	Flow rate (ml/s)	Resistance (mmHg.s/ml)	Compliance ( × 10^−4^)
Sham4	1.01 ± 0.02	0.80 ± 0.03	1.23 ± 0.08	0.96 ± 0.04	122.49 ± 5.86	10.31 ± 0.75
ShamZn4	1.08 ± 0.03	0.93 ± 0.04	1.05 ± 0.07	0.95 ± 0.02	125.20 ± 6.12	9.23 ± 0.69
MI4	1.02 ± 0.02 ^*^ *p > 0.9999*	0.82 ± 0.03 ^*^ *p > 0.9999*	0.48 ± 0.04^*^^*^ *p < 0.0001*	0.38 ± 0.03^*^^*^ *p < 0.0001*	280 ± 22.82^*^^*^ *p < 0.0001*	4.35 ± 0.44^*^^*^ *p < 0.0001*
MIZn4	1.03 ± 0.02 ^◆^ *p = 0.8314* ^#^ *p = 0.9998*	0.84 ± 0.04 ^◆^ *p = 0.8369* ^#^ *p > 0.9999*	0.66 ± 0.05^◆^^◆^ *p = 0.0005* ^#^ *p = 0.2388*	0.55 ± 0.03^◆#^^◆^ *p < 0.0001* ^#^ *p = 0.0169*	196 ± 14.83^◆#^^◆^ *p = 0.0099* ^#^ *p = 0.0008*	5.55 ± 0.42^◆^^◆^ *p = 0.0014* ^#^ *p = 0.2290*
Sham6	1.12 ± 0.02	0.98 ± 0.04	0.93 ± 0.06	0.91 ± 0.05	119.18 ± 9.38	9.85 ± 0.155
ShamZn6	1.10 ± 0.02	0.95 ± 0.03	0.91 ± 0.05	0.87 ± 0.06	134.95 ± 6.93	9.75 ± 1.18
MI6	1.15 ± 0.04 ^*^ *p = 0.9995*	1.05 ± 0.07 ^*^ *p = 0.9804*	0.29 ± 0.04^*^^*^ *p < 0.0001*	0.29 ± 0.02^*^^*^ *p < 0.0001*	339 ± 22.86^*^^*^ *p < 0.0001*	3.60 ± 0.642^*^^*^ *p < 0.0001*
MIZn6	1.09 ± 0.03 ^◆^ *p > 0.9999* ^#^ *p = 0.8324*	0.94 ± 0.06 ^◆^ *p > 0.9999* ^#^ *p = 0.7857*	0.24 ± 0.03^◆^^◆^ *p < 0.0001* ^#^ *p = 0.9997*	0.22 ± 0.02^◆^^◆^ *p < 0.0001* ^#^ *p = 0.9622*	409 ± 38.14^◆#^^◆^ *p < 0.0001* ^#^ *p = 0.0228*	0.96 ± 0.168^◆#^^◆^ *p < 0.0001* ^#^ *p = 0.0010*

All of the data were shown as Mean ± SEM. ^*^ *p* < 0.05, MI vs. Sham; ^◆^ *p* < 0.05, ShamZn vs. MIZn; ^#^ *p* < 0.05, MIZn vs. MI.

**FIGURE 4:**
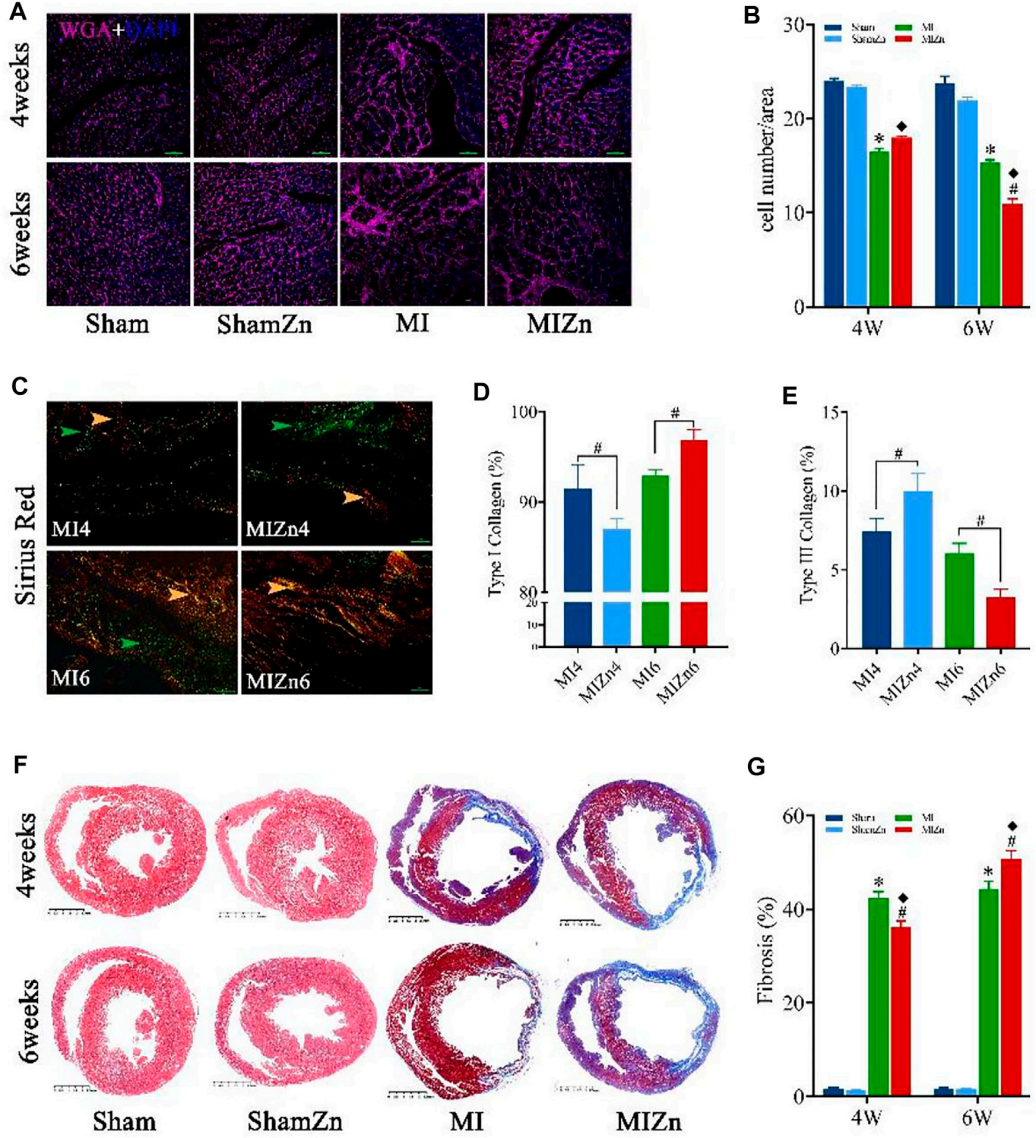
Representative diagrams of WGA + DAPI redyeing **(A)** and statistical results of myocyte number per unit area (image magnification: × 200 and scales: 50 µm) **(B)**; Representative diagrams of Sirius red staining **(C)** and statistical results of type I collagen (yellow) **(D)** and type III collagen (green) **(E)**. Schematic panoramas of Masson staining **(F)** and statistical results of myocardium fibrosis (scales: 2.5 mm) **(G)**. All of the data were shown as Mean ± SEM. ^*^ *p* < 0.05, MI vs. Sham; ^◆^ *p* < 0.05, ShamZn vs. MIZn; ^#^ *p* < 0.05, MIZn vs. MI.

Figures 4A,B show representative diagrams of WGA + DAPI redyeing and statistical results of myocyte number per unit area. The myocyte number per unit area in the MIZn4 group is higher than the MI4 group (18.0 vs. 16.5) with no statistical significance, but that in the MIZn6 group is lower than the MI6 group (11.0 vs. 15.3, *p* < 0.05). Figures 4C–E show representative diagrams of Sirius red staining and statistical results of type I (yellow) and type III (green) collagens. The content of type I collagen in the MIZn4 group is lower than the MI4 group (87% vs. 92%, *p* < 0.05), but that in MIZn6 group is higher than the MI6 group (97% vs. 93%, *p* < 0.05). The content of type III collagen in the MIZn4 group is higher than MI4 group (10% vs. 7%, *p* < 0.05), but that in MIZn6 group is lower than MI6 group (3% vs. 6%, *p* < 0.05). Figures 4F,G show schematic panoramas of Masson staining and statistical results of myocardium fibrosis. Myocardial infarction significantly increases collagens of types I and results in myocardium fibrosis. In comparison with the MI groups, the inhalation of ultrafine Zn particles inhibits the deuteriation of myocardial fibrosis in the MIZn4 group (MI4 vs. MIZn4: 42% vs. 36%, *p* < 0.05), but accelerates the impairments in the MIZn6 group (MI6 vs. MIZn6: 45% vs. 51%, *p* < 0.05).

## Discussion

The present study investigated the changes of cardiac functions and hemodynamics in MI rats at the end of long period of inhaling ultrafine Zn particles. The long-term (6 weeks) inhalation of ultrafine Zn particles is found to impair cardiac functions and hemodynamics in MI rats while the short-term (4 weeks) inhalation has a protective effect.

Zinc has the highest concentration in the trace metals of air pollution in the YRD Metropolitan Area, China (Ming et al., 2017). Exposure to UFPs in air pollution leads to cardiovascular morbidity and mortality (Brook et al., 2010). As a logistic starting point, we investigated the effects of inhaling ultrafine Zn particles in the rat MI model. Monse et al. found that controlled exposures to ZnO nanoparticles caused both airway and systemic inflammations in human subjects, which were observed at a concentration of 0.5 mg/m^3^ and higher (Monsé et al., 2018; Monsé et al., 2019). Since molecular weight of Zn is five times higher than that of O, we selected ultrafine Zn particles (wrapped by a layer of ZnO) of 0.5 mg/m^3^ in the rat model. The Zn level in both serum and heart tissue gradually increased over time after MI rats inhaled ultrafine Zn particles.

Myocardial infarction activated atrial natriuretic peptide, which resulted in an increase of urinary Zn excretion and a decrease of Zn concentrations in plasma and erythrocytes. The significant decrease of Zn concentrations in serum and heart tissue led to myocardial structural and functional disorders (Ripa et al., 1998). The short-term inhalation of ultrafine Zn particles was found to retain the Zn level to a normal range in serum and heart tissue and slow down LV dysfunctions and remodeling in MI rats, consistent with a previous study (Li et al., 2021). On the other hand, there were higher Zn concentrations in the MIZn6 group than others. The higher Zn accumulation is deleterious to MI rats, which accelerates the progression from MI to heart failure. Substantial studies have shown the important role of Zn in redox signaling pathway and antioxidant biological process (Korichneva 2006; Foster and Samman 2010; Maret, 2019), which showed that pro-oxidation due to low or high Zn concentrations is a risk factor for myocardial structural and functional disorders. Excessive Zn concentrations can also inhibit the key enzymes of phosphorylation and redox (Maret, 2019) and induce the inflammation (De Paula et al., 2014).

In the STE analysis, peak myocardial strains and strain rates show the shortening of working myocardial fibers and the relaxation of working myocardial fibers, respectively, which characterize cardiac functions (Niu et al., 2020; Li et al., 2021). The peak values decreased with time after the occurrence of MI in rats. The MI-induced decrease of strains and strain rates denoted the deterioration of systolic and diastolic functions. The short-term inhalation of ultrafine Zn particles inhibited the MI-induced decrease of strains and strain rates while the long-term inhalation accelerated the deterioration of systolic and diastolic functions, which mainly results from myocardium fibrosis. The UFPs-induced change of Zn concentrations affected the content and proportion of types I and III collagen in myocardium of MI rats. Type I collagen is coarse fiber with low ductility and elasticity and high stiffness while type III collagen is fine fiber with mechanical properties opposite to type I collagen. Myocardial infarction stimulated the growth of type I and type III collagen fibers. There was an increase of type III collagens in a week after the MI surgery and then a significant increase of type I collagens after the first week. The increase of type I collagens can create cardiac scarring (Cleutjens et al., 1995) and impair systolic and diastolic functions in MI rats. In comparison with the MI rats, there was lower and higher ratios of type I collagens in MIZn4 and MIZn6, respectively. Matrix metalloproteinases (MMPs) are a family of Zn-dependent endopeptidases involving in the breakdown of extracellular matrix and basement membrane components such as collagen, elastin, fibronectin, gelatin, and laminin (Murphy et al., 1991; Roy and Carey 1997; Page-Mccaw et al., 2007). The decrease of Zn-dependent MMPs is a risk factor for the increase of type I collagens (Tian et al., 2015), which still requires more investigations.

The increased stiffness and resistance in peripheral arteries of MI rats can contribute to the incidence and progression of heart failure (Wu et al., 2017; Huo et al., 2018; Bing et al., 2020; Niu et al., 2020; Li et al., 2021). The MIZn6 group had higher vascular stiffness and resistance than the MI6 group, but the MIZn4 group had the opposite change. The stiffness index in peripheral arteries is associated with the high level of type I collagen in serum (Chatzikyriakou et al., 2014), which was caused by the increased Zn concentrations in serum in the MIZn6 group. Moreover, high Zn concentrations induce oxidative stress and inflammation as well as disrupt the autonomic nervous system (Brook et al., 2010; Cutrufello et al., 2011), which can increase arterial stiffness and resistance. Hence, Zn-regulated changes of arterial stiffness and resistance, in conjunction with cardiac changes, affected the development of heart failure in MI rats.

### Implications of the Study

Previous studies showed the highest concentrations of trace metal Zn, but did not measure its chemical and physical forms in air pollutions in the YRD region, China (Shen et al., 2014; Ming et al., 2017). The emissions of trace metals resulted from various sources, such as vehicle emissions. In order to test the proposed hypothesis, we selected ultrafine Zn particles wrapped by a layer of ZnO. The concentration of Zn is about 193–1,360 ng/m^3^ in air pollutions in China. Human respiratory capacity is about 70–100 times higher than rats. Based on the formula of drug dose conversion between humans and animals, the drug dose of rats should be about 56 times higher than human. Hence, we selected a relative biosafety dose of 500 ug/m^3^, similar to previous studies (Monsé et al., 2018; Monsé et al., 2019). The cardiac and hemodynamic dysfunctions caused by the long-term inhalation of ultrafine Zn particles suggest that we should be looking at ways to lower zinc concentrations in air pollutions, for example, using electric vehicles to reduce the emissions. On the other hand, zinc is a nutrient to improve immune system and metabolism function in human. The results from the short-term inhalation of ultrafine Zn particles suggest that MI patients should obtain enough zinc from food sources of zinc, e.g., chicken, red meat and so on.

### Critique of the Study

This study investigated the toxic effect of ultrafine Zn particles on the progression from MI to heart failure in the rat model. Although cardiac strains and strain rates were analyzed by the STE measurement, myocardial stresses are needed to be studied similar to previous studies (Yin et al., 2020; Zhao et al., 2020). Although we measured blood inflammation factors CRP/IL8/TNF-α and biomarker of heart failure BNP in some animals, which showed that Zn deficiency could increase oxidative stress and promote inflammation in MI rats, the molecular and cellular analysis is still required to find the mechanisms relevant to the effect of Zn. This study only used ultrafine Zn particles with the concentration of 500 ug/m^3^. We will investigate different doses of ultrafine Zn particles as well as other metal constituents in the MI rat model in the following studies.

## Conclusion

Zinc is a main metal component in air pollutions and an essential trace element in the body. The high and low Zn concentrations in serum and tissue have different effects on MI rats. This study carried out the analysis of cardiac functions, histomorphology, and hemodynamics in peripheral arteries in the Sham4, ShamZn4, MI4, MIZn4, Sham6, ShamZn6, MI6, and MIZn6 groups. In comparison with the MI rats, the short-term (4 weeks) inhalation of ultrafine Zn particles supplied the zinc deficiency state and slowed down the progression from MI to heart failure, but the long-term (6 weeks) inhalation induced accumulation of zinc and accelerated the development. Hence, it is of importance for MI patients to regulate Zn intake to slow down the MI progression.

## Data Availability

The original contributions presented in the study are included in the article/supplementary material, further inquiries can be directed to the corresponding author.

## Abbreviations

CO, cardiac output; ESV, end-systolic volume of LV; EDV, end-diastolic volume of LV; EF (%), ejection fraction; FS (%), fractional shortening; HFpEF, heart failure with preserved ejection fraction; HFrEF, heart failure with reduced ejection fraction; LV, left ventricle; LAD, left anterior descending artery; LVAW;s, LV anterior wall in systole; LVAW;d, LV anterior wall in diastole; LVPW;s, LV posterior wall in systole; LVPW;d, LV posterior wall in diastole; LVID;s, LV internal diameter in systole; LVID;d, LV internal diameter in diastole; LVEDP, LV end-diastolic pressure; SV, stroke volume.

## APPENDIX

Histological evaluation: Animals were terminated for histological analysis at postoperative 4 or 6 weeks. After hearts were harvested, plugs of myocardial tissues were removed from different positions of the LV. These plugs were fixed in 4% paraformaldehyde (PFA)/PBS solution overnight at room temperature and processed for paraffin sectioning. Masson’s trichrome and Picro-Sirius Red (PSR) staining was carried out according to standard procedures. Sections were also detected *via* wheat germ agglutinin (WGA) conjugated to Alexa Fluor 594 (50 ug ml^−1^, Invitrogen), similar to a previous study (Wu et al., 2017). Moreover, nuclear morphology was assessed by Hoechst 33,258 dye (Molecular Probes).

Zinc detection: Fresh heart tissue (50 mg) was cut into pieces and dissolved with 65% concentrated nitric acid (1 ml) in a beaker, which was heated for about 30 min until the tissue was completely dissolved. Distilled water with 1 ml 65% concentrated nitric acid was added to the beaker for the 5 ml solution, which was filtrated to obtain the clear liquid. The ICP-OES (iCapRQ, Thermal Fisher Scientific) was used to determine the zinc concentration in the sample solution. The zinc concentration in the serum were also detected using the previous method (Li et al., 2021).
